# A simple method to import CAD mesh format models in FLUKA

**DOI:** 10.1002/acm2.14107

**Published:** 2023-08-10

**Authors:** Sixue Dong, Yinxiangzi Sheng, Jiazhou Wang, Weigang Hu

**Affiliations:** ^1^ Department of Radiation Oncology Fudan University Shanghai Cancer Center Shanghai China; ^2^ Department of Oncology Shanghai Medical College Fudan University Shanghai China; ^3^ Shanghai Clinical Research Center for Radiation Oncology Shanghai China; ^4^ Shanghai Key Laboratory of Radiation Oncology Shanghai China; ^5^ Department of Medical Physics Shanghai Proton and Heavy Ion Center Shanghai China; ^6^ Shanghai Engineering Research Center of Proton and Heavy Ion Radiation Therapy Shanghai China

**Keywords:** CAD mesh model, geometry conversion, Monte Carlo simulation, voxel model

## Abstract

**Background:**

Monte Carlo (MC) code FLUKA possesses widespread usage and accuracy in the simulation of particle beam radiotherapy. However, the conversion from computer‐aided design (CAD) mesh format models to FLUKA readable geometries could not be implemented directly and conveniently. A simple method was required to be developed.

**Purpose:**

The present study proposed a simple method to voxelize CAD mesh format files by using a Python‐based script and establishing geometric models in FLUKA.

**Methods:**

Five geometric models including cube, sphere, cone, ridge filter (RGF), and 1D‐Ripple Filter (1D‐RiFi) were created and exported as CAD mesh format files (.stl). An open‐source Python‐based script was used to convert them into voxels by endowing X, Y, and Z (following the Cartesian coordinates system) of solid materials in the three‐dimensional (3D) grid. A FLUKA (4‐2.2, CERN) predefined routine was used to establish the voxelized geometry model (VGM), while Flair (3.2‐1, CERN) was used to build the direct geometry model (DGM) in FLUKA for comparison purposes. Uniform carbon ion radiation fields 8×8 cm^3^ and 4×4 cm^3^ were generated to transport through the five pairs of models, 2D and 3D dose distributions were compared. The integral depth dose (IDD) in water of three different energy levels of carbon ion beams transported through 1D‐RiFis were also simulated and compared. Moreover, the volume between CAD mesh and VGMs, as well as the computing speed between FLUKA DGMs and VGMs were simultaneously recorded.

**Results:**

The volume differences between VGMs and CAD mesh models were not more than 0.6%. The maximum mean point‐to‐point deviation of IDD distribution was 0.7% ± 0.51% (mean ± standard deviation). The 3D dose Gamma‐index passing rates were never lower than 97% with criteria of 1%–1 mm. The difference in computing CPU time was 2.89% ± 0.22 on average.

**Conclusions:**

The present study proposed and verified a Python‐based method for converting CAD mesh format files into VGMs and establishing them in FLUKA simply as well as accurately.

## 1 INTRODUCTION

Simulation of particle transportation provided great convenience for medical applications, especially radiotherapy, while Monte Carlo (MC) codes are regarded as the gold standard for dosimetry calculation.^1^ Among numerous MC programs, FLUKA (4‐2.2, CERN) was widely used in the simulation of particle therapy for beam model establishment,^2^ beam‐modulated equipment development,^3^ and biological dose calculation,^4^ etc.

Flair (3.2‐1, CERN) provided the graphical user interface (GUI) specifically for FLUKA. Though Flair supported the convenient geometry modeling and error checking in FLUKA, complex geometry models which composed by numerous basic geometry types such as “cube,” “sphere,” and “cone” still required lots of time to establish. Therefore, importing the 3D geometry model which sketched in computer‐aided design (CAD) into FLUKA became a more efficient way for geometry modeling, thus the geometric conversion between FLUKA and CAD during the modeling process would be increasingly necessary, and more simple and efficient methods are required to save the user efforts.

Considering the aforementioned requirement, SimpleGeo had been developed by Theis et al.^5^ to create geometry models based on CAD and visualized with Flair directly. However, the geometry model could only consist by the card types in FLUKA like the Geometry Description Markup Language (GDML) files,^6^ it could not describe the higher‐order surfaces and thus could not describe several geometries such as torus because FLUKA did not support them. Andrew et al.^7^ developed FluDAG (FLUKA integrated with direct accelerated geometry Monte Carlo, DAGMC) to enable FLUKA to transport particles in CAD geometries. However, as an intrinsic tool of FluDAG, CUBIT^8^ is not an open‐source program for public users. Besides, some private programs such as CAD‐PSFO^9^ have also been developed for modeling conversion between FLUKA and CAD.

In this study, we aimed to provide a simple method to import CAD mesh format models in FLUKA by using open‐source codes. Firstly, an open‐source code was used to voxelize CAD mesh format models. Secondly, the voxelized data was converted into FLUKA‐acceptable file format by using an in‐house developed code, and was compiled then imported to FLUKA as the voxelized geometry model (VGM). To validate the accuracy of this method, five types of VGMs and the corresponding direct geometry model (DGM) were established in FLUKA. 1D integral depth dose (IDD) distributions, 2D and 3D dose distributions, geometric volumes, as well as computing speeds were compared. Consequently, further optimization and prospects for the future were discussed.

## 2 METHODS AND MATERIALS

**FIGURE 1:**
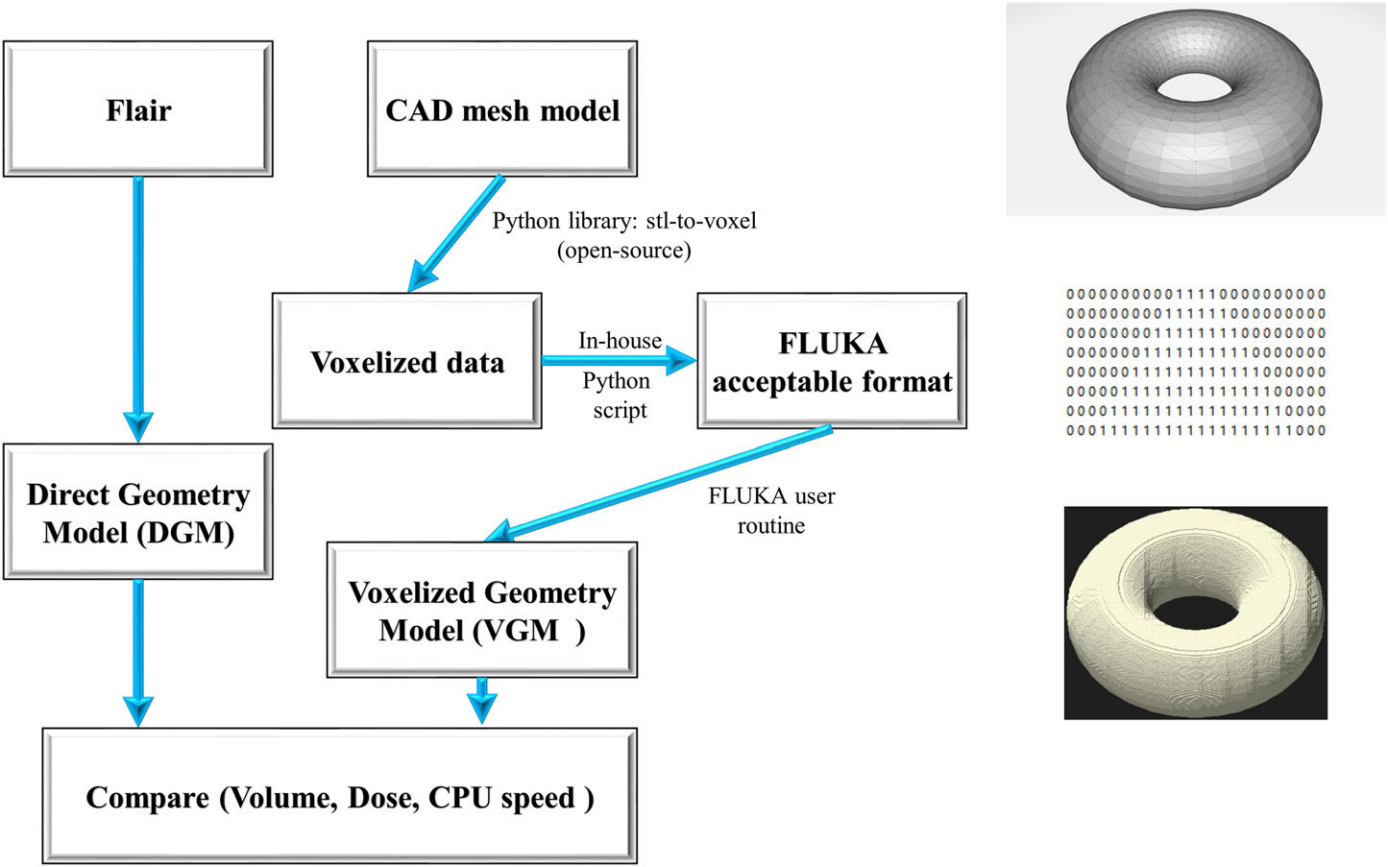
The workflow of this study. FLUKA geometry models were generated from two different methods. The torus model on the right side is an example of the voxelization process.

The workflow of our study is presented in Figure 1. The FLUKA geometry models were generated from the two methods, and the torus model is displayed on the right side as an example of the voxelization process.

### 2.1 Geometry modeling processes

Before the modeling of VGMs, the corresponding CAD mesh models were established by FreeCAD (an open‐source CAD editor, version of 0.20) and exported as “stl (standard triangle language)” format files. Afterward, an open‐source Python (version of 3.11) library “stl‐to‐voxel” (version of 0.9.3)^10^ was used to convert “.stl” format files to 3D space coordinate points files. There were two different numbers “1” and “0” in the 3D matrix array, corresponding to the entity part and the empty space in the 3D grid, respectively. Subsequently, a simple script based on Python (refer to Appendix 1, uploaded simultaneously to GitHub^11^) was used to import 3D spatial coordinate files into 3D matrix arrays, and save as a FLUKA acceptable format (.txt) for further use. According to the FLUKA user manual,^12^ the combination of the user routine “writegolem.f” and the “.txt” file was compiled to generate the “golem” files, a “voxel card” was used to import the “golem” files and build the VGMs in FLUKA. The compiled “golem” files can be visualized in Flair.

Concurrently, the DGMs were constructed by combining fundamental geometric elements utilizing the corresponding geometry cards in FLUKA.

### 2.2 Establish geometry models

**FIGURE 2:**
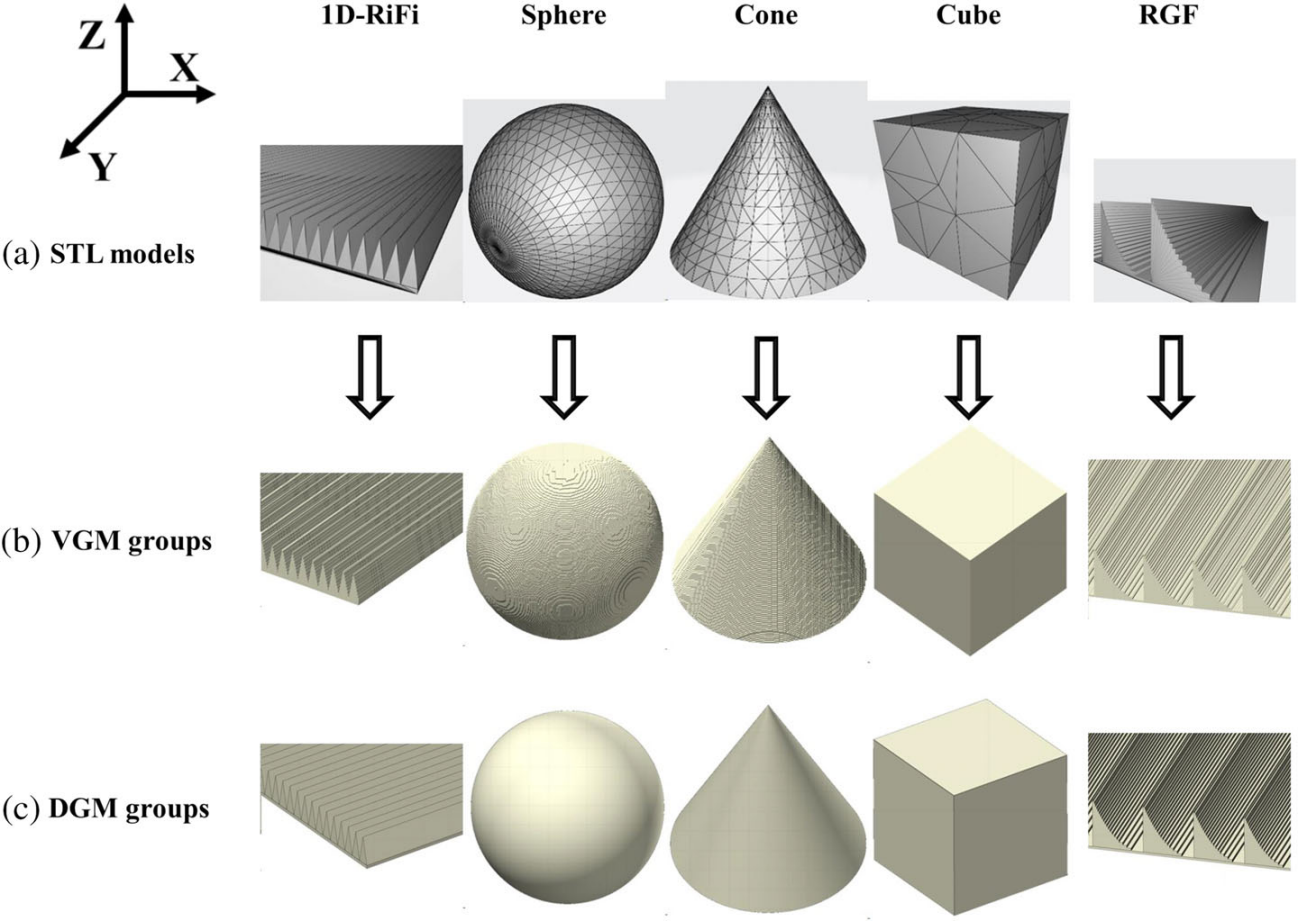
Comparison among the STL models (a), VGMs (b), and DGMs (c). Five types of models (1D‐RiFi, Sphere, Cone, Cube, and RGF) were displayed. DGM, direct geometry model; RGF, ridge filter; VGM, voxelized geometry model.

CAD mesh format models of cube, cone, sphere, ridge filter (RGF),^13^ and 1D‐ripple filter (1D‐RiFi)^14^ were established by FreeCAD and shown in Figure 2a, the size of cube was 10 × 10 × 10 cm^3^, the major radius and height of the cone were 5 and 10 cm, respectively, the radius of the sphere was 5 cm. Regarding the RGF, a total of 21 ridge bar shapes with a cumulative height of 10.4 mm, were systematically optimized and designed, aiming to generate a 10 mm spread‐out Bragg peak (SOBP) of the 45 MeV/u proton beam. For the 1D‐RiFi, it consists of a 0.3 mm base layer and periodic triangular pyramids with 1 mm length and 2.4 mm height. During the process of mesh model importing, the maximum surface deviation and maximum angle deviation were set to 0.1 mm and 30°.

During the voxelization process, convert resolution serves as an optional parameter that influences the accuracy of the voxelized geometric model. A resolution of 100 in the X‐direction represents the geometry model was voxelized to 100 voxels in the X‐direction. In this study, the resolution was set to 400 in X‐, Y‐, and Z‐directions for the CAD mesh models of cube, cone, and sphere. The non‐uniform dimensions voxel was introduced for the 1D‐RiFi and RGF in order to reduce the total voxel numbers, therefore, to save the compiling time, the resolution was set to 1000 in X‐ and Z‐direction, and 10 in the Y‐direction. The five VGMs and five DGMs identical to the CAD mesh models were established in FLUKA and visualized in Flair as shown in Figure 2b,c. The Cartesian coordinate system is also shown in Figure 2.

### 2.3 Validation of geometric models

To facilitate the validation process, two distinct model groups, DGMs, and VGMs, were prepared for the validation process. Polymethyl methacrylate (PMMA) was assigned as the material for DGMs, whereas for the VGMs, PMMA was assigned as the high‐density material for the entity part, and the low‐density material for the empty space was set to air.

Volume comparison was performed between the two model groups. The calculation of the volume of the VGMs, was performed by the number of entity voxels multiplied by the volume of the small voxel. The volumes of the DGMs could be calculated from Flair directly. Furthermore, an additional volume comparison of CAD mesh models and VGMs was also performed. The volume deviation is defined as (Vol_VGM/STL_—Vol_DGM_)/Vol_DGM_.

**FIGURE 3:**
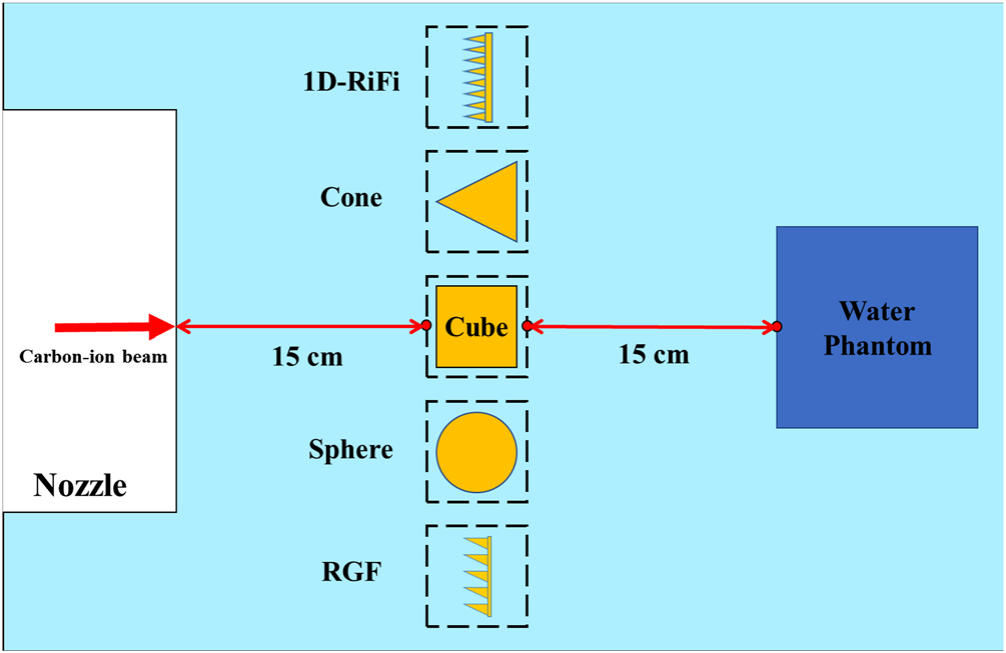
Schematic illustration of the simulation setup. The carbon‐ion/proton beam will pass through the five models successively. RGF, ridge filter.

In this study, we utilized two square homogeneous fields, simulating 4 × 4 cm^2^ and 8 × 8 cm^2^, for the purpose of delivering irradiation in two distinct model groups, the schematic illustration of the simulation setup was depicted in Figure 3. The irradiation procedure involved employing a 430.1 MeV/u carbon ion beam and a 45 MeV proton beam. To ensure consistent positioning, the anterior surfaces of the model groups were placed at a distance of 15 cm downstream from the nozzle. Subsequently, a cubic water phantom with dimensions of 30 × 30 × 30 cm^3^ was positioned downstream from the posterior surfaces of the model groups, also at a distance of 15 cm. The primary objective was to assess the dose distribution within this water phantom, and a spatial resolution of 0.5 mm was employed to record the dose distribution in all directions. The gamma index analysis tool (VeriSoft 7.1, PTW – Freiburg, Germany), which allowed for a comprehensive assessment of the agreement between the calculated and measured dose distributions, was chosen to analyze the 3D dose consistency. The parameters of the gamma analysis were: 2%–2 mm and 1%–1 mm, regions below 10% of the maximum dose would be negligible. and the normalization of gamma analysis was performed on the global dose maximum.^15^, ^16^ In addition, 234.1, 351.4, 430.1 MeV/u carbon ion beams and 45 MeV proton beam were simulated to pass through the two kinds of 1D‐RiFi and RGF models, respectively, and the deposited absorbed doses were also scored in the cubic water phantom with the same scoring grid as above. The corresponding IDDs were simulated with a 4.08 cm radius of the detection region in the FLUKA subsequently.

The computing system in the simulation work is the same as the configuration used by Sheng et al.^17^ in establishing the treatment nozzle for the raster scanning proton beam. One host and three nodal servers (with a total CPU number of 112) were chosen as the work platform, and the LINUX shell script was used to execute the input file to achieve the purpose of parallel computing. Taking a point source of 234.05 MeV/u carbon ion beam as an example, 1 × 10^8^ primary particles were required to maintain the statistical error below 1% for the volumes with dose above 5% of the maximum dose. The default setting of “HADROTHErapy” was used in the FLUKA for particle transportation. For the monoenergetic carbon ion beam of 430.1 MeV/u, 1 × 10^9^ primary particles were set for all the input files and the CPU calculation time between DGMs and VGMs were recorded and compared. The deviation of CPU calculation time is defined as (T_VGM_—T_DGM_)/T_DGM_.

## 3 RESULTS

### 3.1 Volume comparison

After the conversion, take the VGM of cube as an example, the voxel dimensions were 0.25125, 0.25125, and 0.25125 mm, while for the VGM of 1D‐RiFi, the lengths of the voxel were 0.06006, 6.667, and 0.002703 mm in X‐, Y‐, and Z‐directions, respectively. The detailed geometric parameters and comparison of STLs, DGMs, and VGMs were shown in Table 1. The maximum deviation between STLs and DGMs was 0.57%, moreover between VGMs and DGMs was 0.60%, respectively.

**TABLE 1:** Geometric parameters and comparison between STLs, DGMs, and VGMs (two valid numbers are retained).

				STL Vol (cm^3^)	Vol_STL_ deviation
	Voxel vol (mm^3^)	Voxel number	DGM Vol (cm^3^)	VGM Vol (cm^3^)	Vol_VGM_ deviation
1D‐RiFi	0.0011	2.0 × 10^7^	21.60	21.60	0.00%
				21.47	0.60%
Cube	0.016	6.3 × 10^7^	1000.00	1000.00	0.00%
				1004.34	0.43%
Cone	0.016	1.6 × 10^7^	261.80	260.58	0.47%
				261.04	0.29%
Sphere	0.016	3.3 × 10^7^	523.60	520.60	0.57%
				524.26	0.13%
RGF	0.017	3.0 × 10^7^	50.06	49.95	0.22%
				49.98	0.16%

Abbreviations: DGM, direct geometry model; RGF, ridge filter; VGM, voxelized geometry model.

### 3.2 Gamma passing rates (γ‐PRs) and IDDs

**FIGURE 4:**
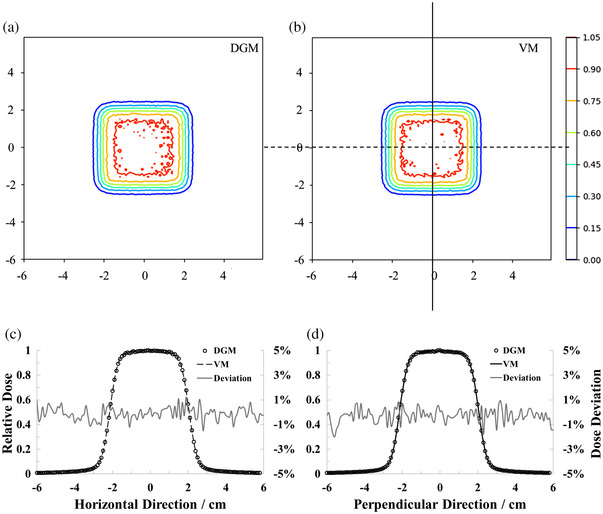
Comparison of 2D and 1D dose distributions between the 4 × 4 cm^2^ plane of the 430.1 MeV/u carbon ion Bragg peak location perpendicular to the beam. (a) Was modulated by DGM‐1D‐RiFi, (b) was modulated by VGM‐1D‐RiFi, and (c,d) were 1D dose profiles extracted from the 2D dose distributions. Point‐to‐point dose deviations were also shown at the subordinate axis. DGM, direct geometry model.

The 3D γ‐PRs between VGMs and DGMs with were shown in Table 2. All γ‐PRs could achieve 97.0% with the criterion of 1%–1 mm, and almost 100% with the criterion of 2%–2 mm. Figure 4a,b presented the 2D dose distributions of the 1D‐RiFi group at the plane of the Bragg peak location vertical to beam line as an example, 1D dose profiles extracted from the dashed (c) and solid line (d) of the case were also displayed. The mean absolute point‐to‐point deviations^18^ of the 1D dose distribution were 0.1% ± 0.5% (mean ± standard deviation, SD) and 0.28% ± 0.58% in the horizontal direction and the perpendicular direction, respectively. The influence of field size was less than 1% on the results of the γ‐PRs.

**TABLE 2:** 3D γ‐PRs between VGMs and DGMs with criteria of 2%–2 mm and 1%–1 mm.

	2‐2		1‐1	
	4 × 4	8 × 8	4 × 4	8 × 8
1D‐RiFi	100.0%	99.9%	97.9%	97.9%
Cube	100.0%	99.8%	97.5%	97.2%
Cone	100.0%	99.8%	97.6%	97.4%
Sphere	100.0%	99.8%	97.2%	97.2%
RGF	100.0%	99.9%	97.8%	97.7%

Abbreviations: RGF, ridge filter.

**FIGURE 5:**
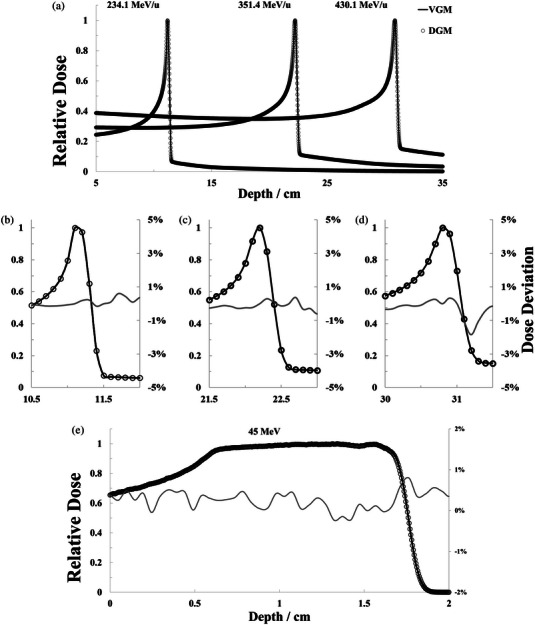
(a) IDD comparison between using DGM‐1D‐RiFi (dotted line) and VGM‐1D‐RiFi (solid line) at different carbon ion energy levels. (b,c,d) Amplified the locations around the peak at 234.1, 351.4, and 430.1 MeV/u carbon ion beam, respectively. (e) Showed the SOBP comparison between using DGM‐RGF (dotted line) and VGM‐RGF (solid line). Point‐to‐point dose deviations were also shown at the subordinate axis in (b), (c), (d), and (e). DGM, direct geometry model; VGM, voxelized geometry model.

For the 1D‐RiFi, the IDD comparison between VGMs and DGMs was shown in Figure 5a, and enlarged in Figure 5b–d to observe the fine discrepancy at the beam range (defined at the distal 80% dose level, R80), the distal falloff width (defined as the distance along the beam axis where the dose in water reduces from 80% to 20%, DFW) and the Bragg peak width (defined as the distance in water between the proximal and distal 80% dose level, BPW).^19^ From the result, the difference of R80 never exceeded 0.005 mm at the three simulated energy levels. The maximum absolute deviation of the BPW and DFW was 0.02 mm/0.5% and 0.05 mm/1.8% which was at the energy level of 430.1 MeV/u. In regions above 20% of the maximum dose, among the three simulated energy levels, the maximum mean dose deviation was 0.7% ± 0.51% (351.4 MeV/u) at the dose region which higher than 5% of the maximum dose. As shown in Figure 5e, the SOBP of 45 MeV/u proton beam was modulated by RGF, between VGM and DGM, the mean dose deviation was 0.28% ± 0.36%, and deviation of SOBP width (defined as the distance in water between the proximal and distal 90% dose level)^19^ was 0.05 mm.

### 3.3 CPU time

When 1 × 10^9^ primary particles were performed, the total simulation time were recorded in Table 3. By using the VGMs, the CPU calculation time was comparable (2.89% ± 0.22% mean ± SD) between using the DGMs, there was no additional burden need to be borne by the computer.

**TABLE 3:** CPU calculation time of DGMs and VGMs, and the deviation between them.

	T_VGMs_ (h)	T_DGMs_(h)	Deviation
1D‐RiFi	63.75	62.12	2.62%
Cube	69.33	67.50	2.75%
Cone	67.40	65.28	3.25%
Sphere	69.58	67.55	3.01%
RGF	64.05	62.27	2.86%

Abbreviations: RGF, ridge filter.

## 4 DISCUSSION

In order to develop a simple method to import CAD mesh format models in FLUKA, voxelization of mesh model were proposed in this study. Three basic geometric models (cube, cone, and sphere) and two fine geometric models (1D‐RiFi and RGF) were established for the purpose of confirming the conversion consistency. By performing the comparison of model volumes and dose distributions between using VGMs and DGMs, the excellent accuracy of the application was validated successfully.

The conversion consistency positively correlated with the resolution. During the process of conversion in this work, 1D‐RiFi and RGF were endowed a high resolution to construct their numerous periodic groove and ridge bar structures. From the calculation results of the Python script, the three side lengths of the voxel were not the same ordinary integers for all geometries, but the similar infinite decimals for especially irregular geometry, so cuboid voxels were usually produced instead of the cube voxels (III.A). When converting a specific region, such as the apex area of a cone, within the DGM into an STL model, followed by the generation of a VGM consisting of cubic voxels, it was unavoidable that certain complex structures might not be replicated with absolute precision. The maximum surface deviation and maximum angle deviation mesh during the process of STL model exporting from FreeCAD was presumed to be the major sources of the volume difference between the DGMs and STL models, because the non‐smooth surfaces of the “stl” format model were fabricated by triangular envelope loops. While the converting resolution, which determined the size and number of voxels, was considered to be the primary factor contributing to the observed differences in volume between the STL model and VGM. Overall in this study, by calculating the volumes among STL models, DGMs, and VGMs, excellent consistency (difference≤ 0.6%) could be achieved for all the models as is shown in Table 1.

The 3D γ‐PRs of all the cases >97% with the criterion of 1%–1 mm. According to the aforementioned discussion, the boundary of a complex geometry with non‐smooth surfaces might lead to relatively prominent difference compared to the DGMs in FLUKA. The difference becomes evident in the form of dose deviations observed in the modulated beams when they are deposited in water. Therefore, a larger radiation field with the size of 8 × 8 cm^2^ was simulated to shoot through the models, and the more obvious influence of marginal differences would be expected. However, almost no discrepancy could be observed when implementing a larger field from the results of γ‐PRs in Table 3. Furthermore, in order to investigate the beam properties when passing through different models, IDDs of three carbon ion energy levels modulated by 1D‐RiFi were recorded and 1D profiles were displayed to compare the BPW, DFW, and R80. From the 1D profiles, the VGM‐1D‐RiFi modulated curves well matched (ΔR80 ≤ 0.005 mm, ΔBPW ≤ 0.5%, ΔDFW ≤ 1.8%) with which modulated by the DGM‐1D‐RiFi around the region of Bragg peak. Apart from this, RGF, another complex geometric model was also established to do the comparison, and the results showed that the IDD curve and SOBP width were both replicated with great precision when VGM was employed instead of DGM. Generally, excellent consistency of contour and shape so far as to the geometry margin could be inferred anyway. Computational efficiency is a crucial factor that significantly affects convenience and should be taken into consideration. In our study, we evaluated the CPU calculation times for both the DGMs and VGMs. The results indicate that the computational times between the two methods were comparable. Therefore, adopting VGMs instead of DGMs would not impose any additional burden on the computer system.

There were three main methods to establish computer‐readable geometry, boundary representation (BREP), constructive solid geometry (CSG), and tessellated polygons. Among them, CSG was provided to FLUKA to simulate geometries with Boolean operations between basic geometries to compose a complex geometry.^20^ Nevertheless, this approach imposes certain limitations on FLUKA due to its reliance on predefined basic geometries, in other words, FLUKA could only do “combination” but not “create.” Consequently, certain complex geometries, such as a torus, cannot be constructed if the requisite basic geometry is unavailable. As a kind of tessellated polygons, “stl” format model was performed to be the object for converting in the present study. Theoretically, numerous triangles could flexibly fabric/create almost all complex finite geometries, and these geometries could be voxelized and established in FLUKA by using our method (see the torus in Figure 1 as an example). When dealing with intricate objects consisting of multiple elementary geometries, converting from DGM to VGM essentially involves using an extensive array of planar structures to accurately represent the curved surface structures, consequently, it was unavoidable that edge distortion will occur. A VGM is generally composed of millions of micron voxels. Smaller voxel volume results in a higher converting resolution of the CAD mesh format models which could lead to enhanced surface details, and consequently reduce the differences between DGM and VGM especially in the edge area. Users could modify the volume of the VGM (whether proportionally or not) by only changing the size of voxels in the FLUKA user routine file, and simultaneously precheck the 2D as well as 3D viewgraphs of the VGM in Flair. With the development of 3D printing, more and more radiation therapy centers use this technology to materialize the beam‐modulated equipment^21^, ^22^ or human tissue.^23^, ^24^ In our current study, various factors potentially influenced the observed discrepancies across the design→ simulation validation→ 3D printing process for the models. These factors included the maximum surface and angle deviation during STL model export, the resolution employed for the conversion from the STL model to VGM, and the inherent systematic errors and printing accuracy associated with the 3D printer during the printing process. Consequently, mitigating the exporting deviation, improving the conversion resolution, and utilizing a high‐accuracy 3D printer could effectively address the inter‐model variations when feasible. Overall, as the mainstream format of 3D printing, the conversion between STL geometry and FLUKA geometry during the model‐establishing process would become meaningful.

The aim of our work is to voxelize the STL format and import it to FLUKA, models that were assigned by more than two types of materials were not considered during the process of voxelization and modelization, and only two materials were assigned to the binary voxel model. Actually, human organs, mechanical structures composed of various components, or even actual buildings required various materials to do the modeling process.^25^, ^26^ From the commands of our current script shown in the Supplement material, the length of the matrix in x‐, y‐ and z‐directions was obtained according to the conversion resolution for a single STL model adaptively, while the combination STL models with different materials could be achieved by using a matrix which was large enough to accommodate all of the voxels. Additionally, various materials were supported to be assigned to the “voxel card” in FLUKA, the voxel number of the “txt” file only needed to be designated sequentially to represent other materials. Simple and convenient are the prominent advantages that are required to be emphasized eventually, the complete conversion just relies on the Python script/library and the FLUKA user routine file. Compared to other CAD‐MC code conversion methods, no additional programs were employed throughout our study to avoid unnecessary bugs, and possessed the same high accuracy as the other methods. We believed that the method of “conversion by voxelization” could save the time of modeling and user effort significantly. Another point would be mentioned, there were many libraries and/or scripts for the conversion process, and “stl‐to‐voxel” was just one of them. Other scripts such as “obj2voxel” based on C++ could convert “obj” format files to voxels,^27^ and its effectiveness awaited for confirmation.

## 5 CONCLUSION

The present study proposed and verified an open‐source and Python‐based method for converting CAD mesh (STL) format files into VGMs and establishing the geometry models in FLUKA simply as well as accurately. This method could not only simulate a single‐material geometry to provide service for 3D printing, but also had the potential to simulate complex multi‐materials models.

## AUTHOR CONTRIBUTION

Sixue Dong: Responsible for geometry modeling, MC simulation, data analysis, and manuscript writing. Yinxiangzi Sheng: Contributed to describing the initial idea, designing the research, and revising the paper. Jiazhou Wang: Provided critical feedback on the initial manuscript. Weigang Hu: Devised the project outline and provided writing guidance. All authors have thoroughly reviewed and granted final approval of the written manuscript. Each author has concurred to take responsibility for the accuracy and integrity of the results presented within the manuscript.

## CONFLICT OF INTEREST STATEMENT

The authors declare no conflicts of interest.

## Supporting information

Supporting information

## Data Availability

The data that support the findings of this study are available from the corresponding author upon reasonable request.
