# Supplementary material for: A simple method to import CAD mesh format models in FLUKA
Source: J Appl Clin Med Phys. 2023 Aug 10;24(11):e14107. doi: 10.1002/acm2.14107 (PMC10647971; doi:10.1002/acm2.14107)
Supplement: Supplementary file 1 — Supporting information [file ACM2-24-e14107-s001.docx]

**Appendix 1** a simple script based on python that used to import 3D spatial coordinate files into 3D matrix arrays, and save as a FLUKA acceptable format (.txt). The GitHub link is as follows: (https://github.com/DongSixue/STL_to_FLUKA.git)

# -*- coding:utf-8 -*-

import numpy as np

# Load data from file

data = np.loadtxt(r'your\path\to\file.xyz')

# Extract each column and calculate the minimum value and resolution

x, z, y = data[:, 0], data[:, 1], data[:, 2]

# Calculate the minimum values and resolutions for each axis

xmin, xmax = np.min(x), np.max(x)

ymin, ymax = np.min(y), np.max(y)

zmin, zmax = np.min(z), np.max(z)

resoX = np.mean(np.diff(np.sort(np.unique(x))))

resoY = np.mean(np.diff(np.sort(np.unique(y))))

resoZ = np.mean(np.diff(np.sort(np.unique(z))))

print("Resolution:", resoX, resoY, resoZ)

print("Volume:", resoX * resoY * resoZ)

# Transform the coordinates to start from 1 with interval 1

X_new = (x - xmin) / resoX + 1

Y_new = (y - ymin) / resoY + 1

Z_new = (z - zmin) / resoZ + 1

# Calculate the dimensions of the matrix

lenx = int(round(np.max(X_new), 0)) + 1

leny = int(round(np.max(Y_new), 0)) + 1

lenz = int(round(np.max(Z_new), 0)) + 1

print("Matrix dimensions:", lenx, leny, lenz)

print("Coordinate arrays:", X_new, Y_new, Z_new)

# Create a new matrix filled with zeros

OriMar = np.zeros((lenz, lenx, leny))

# Assign the corresponding points to the new matrix and set them to 1

for i in range(len(X_new)):

xx, yy, zz = int(X_new[i]), int(Y_new[i]), int(Z_new[i])

OriMar[zz, xx, yy] = 1

# Reshape the matrix to a 2D matrix

m = OriMar.reshape(lenz * lenx, leny)

np.savetxt(

r'your\path\to\file.txt',

m, fmt='%d'

)
